# Evidence for overdispersion in the distribution of malaria parasites and leukocytes in thick blood smears

**DOI:** 10.1186/1475-2875-12-398

**Published:** 2013-11-06

**Authors:** Imen Hammami, André Garcia, Grégory Nuel

**Affiliations:** 1, Laboratoire de Mathématiques Appliquées (MAP5) UMR CNRS 8145Université Paris Descartes, Paris, France; 2 Institut de Recherche pour le Développement, UMR 216 Mère et enfant face aux infections, tropicales, Université Paris Descartes, Paris, France; 3, Faculté de Pharmacie, Université Paris Descartes, Paris, France

**Keywords:** Parasite and leukocyte counts per HPF, Poisson distribution, overdispersion, negative binomial distribution, mixture models, HMMs, EM algorithm, AIC, BIC, Ordinary pseudo-residuals

## Abstract

**Background:**

Microscopic examination of stained thick blood smears (TBS) is the gold standard for routine malaria diagnosis. Parasites and leukocytes are counted in a predetermined number of high power fields (HPFs). Data on parasite and leukocyte counts per HPF are of broad scientific value. However, in published studies, most of the information on parasite density (PD) is presented as summary statistics (e.g. PD per microlitre, prevalence, absolute/assumed white blood cell counts), but original data sets are not readily available. Besides, the number of parasites and the number of leukocytes per HPF are assumed to be Poisson-distributed. However, count data rarely fit the restrictive assumptions of the Poisson distribution. The violation of these assumptions commonly results in overdispersion. The objectives of this paper are to investigate and handle overdispersion in field-collected data.

**Methods:**

The data comprise the records of three TBSs of 12-month-old children from a field study of *Plasmodium falciparum* malaria in Tori Bossito, Benin. All HPFs were examined systemically by visually scanning the film horizontally from edge to edge. The numbers of parasites and leukocytes per HPF were recorded and formed the first dataset on parasite and leukocyte counts per HPF. The full dataset is published in this study. Two sources of overdispersion in data are investigated: latent heterogeneity and spatial dependence. Unobserved heterogeneity in data is accounted for by considering more flexible models that allow for overdispersion. Of particular interest were the negative binomial model (NB) and mixture models. The dependent structure in data was modelled with hidden Markov models (HMMs).

**Results:**

The Poisson assumptions are inconsistent with parasite and leukocyte distributions per HPF. Among simple parametric models, the NB model is the closest to the unknown distribution that generates the data. On the basis of model selection criteria AIC and BIC, HMMs provided a better fit to data than mixtures. Ordinary pseudo-residuals confirmed the validity of HMMs.

**Conclusion:**

Failure to take overdispersion into account in parasite and leukocyte counts may entail important misleading inferences when these data are related to other explanatory variables (malariometric or environmental). Its detection is therefore essential. In addition, an alternative PD estimation method that accounts for heterogeneity and spatial dependence should be seriously considered in epidemiological studies with field-collected parasite and leukocyte data.

## Background

Microscopy of thick blood smears (TBSs) is the usual and most reliable diagnostic test for *Plasmodium falciparum* malaria [1-7]. Parasite density (PD) is classically defined as the number of asexual parasites relative to a microlitre of blood. PD is assessed either by counting parasites in a predetermined number of high power fields (HPFs), or by counting parasites according to a fixed number of leukocytes. Most of PD estimation methods assume that the distribution of the thickness of the TBS, and hence the distribution of parasites and leukocytes within the TBS, is homogeneous; and that parasites and leukocytes are evenly distributed in TBSs, and thus can be modelled through a Poisson-distribution [1,8-10]. PD data-based inferences also rely on such assumptions [11-17].

Identifying the distribution of parasite and leukocyte data on TBSs is the key to an appropriate analysis. Raghavan [18] recognized that parasites may be missed due to the random variation within a slide. He used the binomial distribution to estimate the probability of missing a positive slide, when only a fixed number of HPFs is read. He assumed that parasites were randomly distributed in the blood film, and that each parasite has the same chance of occupying any of the HPFs read. Dowling & Shute [19] showed that leukocytes are evenly distributed in thick films, and that their number varies directly according to the thickness of the smear. They indicated a normal distribution of leukocytes per HPFs. In addition, they claim that parasites are also distributed evenly throughout the thick blood smear. However, they noticed, in the case of scanty parasitaemia, a phenomenon of “grouping”, in which parasites tend to aggregate together in a specific area of the smear. Petersen *et al.*[9] claimed that estimating the PD from the proportion of parasite-positive HPFs, instead of counting parasites in each field, underestimates the PD in TBSs, since a parasite-positive field may contain more than one parasite. To get ride of this problem, they suggested a correction of the estimation method. Their model was built under the assumption that parasites are Poisson-distributed on the TBSs. Under this assumption, the estimate of the mean number of parasite per field (*λ*) is then $\hat{\lambda }=-\log(1-p)$, where *p* is the percentage of parasite-positive HPFs. However, due to the clustering of parasites in TBSs, $\hat{\lambda }$ was corrected by a factor of 2. This factor of two was empirically chosen without a clear analytical proof. Bejon *et al.*[1] used the Poisson distribution to calculate the likelihood of sampling a parasite within the blood volume examined in microscopy. Alexander *et al.*[20] described the variation across the sample by a homogeneous Poisson distribution of parasites on TBSs. They unpacked -under the Poisson assumption- similar results to Raghavan’s -under the Binomial assumption- at low densities, but he argued for the evidence of discrepancy as density increases.

Two assumptions specific to the Poisson model have been identified as sources of misspecification. The first is the assumption that variance equals the mean. The second is the assumption that events occur evenly. That assumption preludes, for instance, that occurrences in a field influence the probability of occurrences in neighbouring fields. But this type of contagion is to be suspected in the distribution of parasites and leukocytes in TBS. Violations of both assumptions lead to the same symptom: a violation of the Poisson variance assumption. Overdispersion, or extra-Poisson variation, denotes a situation in which the variance exceeds the mean. Unobserved heterogeneity and positive contagion lead to overdispersion [21-24]. Undetected heterogeneity may entail important misleading inferences, so its detection is essential.

Three lines of research exist to account for overdispersion. Firstly, an overdispersion test is helpful, since the lack of significance in testing overdispersion might indicate that a further investigation of latent heterogeneity might not be necessary. Various tests for detecting overdispersion have been developed [25-29]. Secondly, the effect of overdispersion has been analysed and corrected within the maintained Poisson model [9,30]. Thirdly, various models have been proposed that account for unobserved heterogeneity while nesting the Poisson model as a special case [31-38]. Standard approaches employ mixture distributions, either parametrically by introducing models that accommodate overdispersion, for example the negative binomial models, or semiparametrically by leaving the mixing distribution unspecified [9,39]. These parametric and semiparametric models involve an extra-dispersion parameter, which requires numerical methods for its estimation [40-42].

In published studies, malariological data are presented as summary statistics (e.g. parasite density per microlitre, prevalence, absolute or assumed WBC count). Parasite and leukocyte counts per field, while of great importance, are not available in the open literature or in archived sources. A dataset of parasite and leukocyte counts per HPF was then constituted and published in this study. Three TBSs of 12-month-old children were entirely examined. All HPFs were read sequentially. The number of parasites and the number of leukocytes per HPF were recorded. The aim of this study is twofold: to examine the presence of overdispersion in the distribution of parasites and leukocytes in TBSs, and to fit the appropriate model that allows for overdispersion in these data. To do so, two sources of overdispersion are explored: the latent heterogeneity in parasite and leukocyte counts, i.e. the presence of homogeneous zones (where the data have a similar distribution) associated to an unobserved state, and the spatial dependence in data, i.e: the correlation between neighbouring occurrences.

## Materials and methods

### Epidemiological data

**Figure 1:**
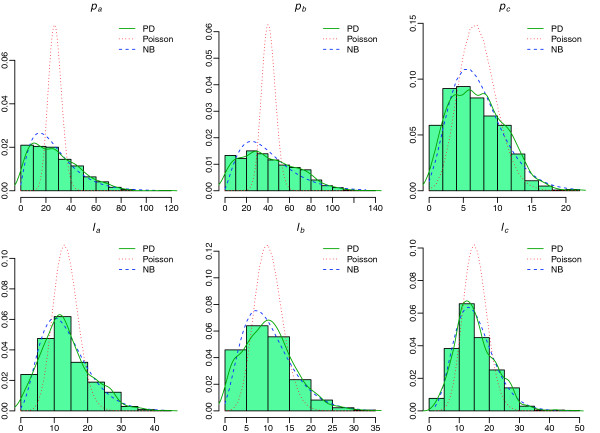
***Histograms of parasite and leukocyte counts per HPF***. The empirical density function and the fitted distributions (Poisson, NB) are displayed on the top of each histogram.

The data accompanying this study were gathered from a field study of *Plasmodium falciparum* malaria in the district of Tori Bossito located 40 km North-East of Cotonou, South Benin. Across this field study, 550 infants were followed weekly from birth to 12 months [43,44]. Malaria is perennial in the study area, and according to a recent entomological survey *P. falciparum* is the commonest species (95%), *Plasmodium malariae* and *Plasmodium ovale* representing respectively 3% and 2% [45]. From the *Tori-Bossito* study, three thick films of 12-month-old children were randomly selected among positive slides and included in this study. TBSs were stained with Giemsa. All high power fields (HPFs), defined as oil immersion microscopic fields (×1,000), were re-examined by visually scanning the entire film horizontally from edge to edge. The number of parasites (*p*) per field and the number of leukocytes (*ℓ*) per field were derived. The letters “*a*”, “*b*” “*c*” denote the three selected TBSs throughout this paper. A summary of the data is given in Table (1). Histograms of the data are plotted in Figure 1 in order to help for visualizing the shape of the data before the distributions are fitted. The full dataset can be found in Additional file 1.

**Table 1:** Descriptive statistics of parasite and leukocyte counts on TBSs

TBS	***a***		***b***		***c***	
Number of HPFs	754		938		836	
Volume of blood^***∗***^ (*μ* ***l***)	1.51		1.88		1.67	
PD ^***‡***^** (parasites/*μ*** ***l***)	16,190.79		31,783.18		3,725.95	
**Parasites and**	***p***_***a***_	***ℓ***_***a***_	***p***_***b***_	***ℓ***_***b***_	***p***_***c***_	***ℓ***_***c***_
**leukocytes**						
Total number	20621	10189	38112	9593	5989	12859
Mean (per HPF)	27.35	13.51	40.63	10.23	7.16	15.38
Median	25	13	37	10	7	14
Range	0-111	0-43	0-131	0-35	0-22	2-47
IQR ^***†***^	12-40	8-17	20-60	6-14	4-10	11-19
Standard deviation	18.76	7.22	25.94	5.90	3.92	6.62
% negative ^***§***^	1.06	1.06	0.75	1.39	1.08	0.00

Three thick blood smears are studied “***a*”, “*b*”, “*c*”.**

Parasite and leukocyte counts for each TBS are denoted (***p***_***a***_**,** ***ℓ***_***a***_**), (*p***_***b***_**,** ***ℓ***_***b***_**) et (*p***_***c***_**,** ***ℓ***_***c***_**).**

^*^Assuming that the volume of blood in one HPF is approximately 0.002 ***μ*** **l [**[19,46,47].

^**‡**^**PD**$=\frac{p}{\ell }\times$** 8,000, assuming that the number of leukocytes per microlitre of blood is 8,000 [**[7,48,49].

^**†**^Inter-Quartile Range.

^**§**^Percentage of negative high-power fields (HPFs) where no parasites and/or no leukocytes are seen.

### Statistical models for parasite and leukocyte data

Some laboratory counting techniques consist in reading a certain volume of blood (say ***u*** ***μ*** ***l*) before the film is declared negative. If parasites are seen in** ***u*** ***μ*** ***l*, then an additional volume (say** ***v*** ***μ*** ***l*) is read. The volume of blood contained in one HPF is approximately 0** ***.*** **002** ***μ*** ***l*** [19,46,47]. The assumed number of white blood cells per microlitre of blood is 8,000 [7,48]. In practice, ***u*** ***μ*** ***l* may correspond to 100 HPFs (i.e.** ***u*** **=0** ***.*** **2** ***μ*** ***l*), and** ***v*** ***μ*** ***l* may correspond to 200 white blood cells (i.e.** ***v*** **=0** ***.*** **025** ***μ*** ***l***) [7,50-52]. In this example, parasites are assumed to be spread evenly throughout the TBS with density ***θ*** ***μ*** ***l*. Under the Poisson assumption, the probability of seeing no parasites in** ***u* volume of blood is** ***e***^-***θ*** ***u***^**, and the probability of seeing exactly** ***x* parasites (*x*** **>0) is then (1-*e***^***θ*** ***u***^**)** ***e***^**-*θ*** ***v***^**(*θ*** ***v*)**^***x*-1**^/(***x*-1)!. The latter probability is the product of the probability of seeing at least one parasite in volume** ***u*, and the probability of seeing (*x*-1) more parasites in volume** ***v*. Under this procedure, the estimation of the PD depends on volumes** ***u* and** ***v***, which are not the same for all slides.

The restrictive nature of the equidispersion assumption in the Poisson model led to the development of numerous techniques both for detecting and modelling overdispersion [25,26,28,31,53-55]. This section details alternative models used to fit the PD and leukocyte data.

#### Simple parametric models

The typical alternative to the Poisson model is the negative binomial (NB) model, which is an attractive model that allows overdispersion. The dispersion parameter ***ϕ*** in the NB controls the deviation from the Poisson. This makes the NB distribution suitable as a robust alternative to the Poisson. However, it is useful to obtain more general specifications through other modelling frameworks that handle overdispersion or zero-inflation (NB, geometric, logistic, Gaussian, exponential, zero-inflated Poisson (ZIP), Poisson hurdle (HP), zero-inflated negative binomial (ZINB), negative binomial hurdle (HNB)). The main motivation behind using zero-inflated [56,57] and hurdle count models [35,58] is that PD data frequently display excess zeros at low parasitaemia levels. Zero-inflated and hurdle count models provide a way of modelling the excess zeros in addition to allowing for overdispersion. These models include two possible data generation processes (one generates only zero counts, whereas the other process generates counts from either a Poisson or a negative binomial model).

#### Finite mixture models

One method of dealing with overdispersed observations with a bimodal or more generally multimodal distribution is to use a finite mixture model. Mixture models are designed to account for unobserved heterogeneity in a set of data. The sample may consist of unobserved groups, each having a distinct distribution for the observed variable. Consider for example the distribution of parasites per HPF, ***X***_***t***_**. The fields can be divided into groups according to its locations, e.g. edges and center of the film. Even if the number of parasites within each group was Poisson-distributed, the distribution of** ***X***_***t***_** would be overdispersed relative to the Poisson. In the case of a two-component mixture with weights (*δ***_**1**_**,** ***δ***_**2**_**), means (*λ***_**1**_**,** ***λ***_**2**_**) and variances**$\left(\sigma_{1}^{2},\sigma_{2}^{2}\right)$**, the total variance exceeds the mean by** ***δ***_**1**_ ***δ***_**2**_**(*λ***_**1**_**-*λ***_**2**_**)**^**2**^ (details of the proof are given in Additional file 2). Hence, the two-state Poisson mixture is able to accommodate overdispersion better than the Poisson model with one component. The mixture component identities are defined by some latent variables (also called the ***parameter process*). If the latent variables are independent, the resulting distribution is called** ***independent* mixture. An independent mixture distribution consists of a finite number, say** ***m***, of component distributions and a mixing distribution which selects from these components. Note, however, that the above definition of mixture models ignores the possibility of spatial dependence in data, a point that shall be addressed by introducing Hidden Markov Models (HMMs), which connect the latent variables into a Markov chain instead of assuming that they are independent.

#### Hidden Markov models (HMMs)

Unlike the mixture models, where observations are assumed independent of each other and the spatial relationship between neighbouring data is not taken into account, HMMs incorporate this spatial relationship, and show promise as flexible general purpose models to account for such dependency [59-61]. HMMs can be used to describe observable events that depend on underlying factors, which are not directly observable, namely the ***hidden states*. A HMM consists of two stochastic processes: an invisible process of hidden states, namely the** ***hidden process* (also called the** ***parameter process*), and a visible process of observable events, namely the** ***observed process* (or the** ***state-dependent process***). The hidden states follow a Markov chain, in which, given the present state, the future is independent of the past. Modelling observations in these two layers, one visible and the other invisible, is very useful to classify observations into a number of classes, or clusters, and to incorporate the spatial-dependent information among neighbouring observations. In the context of parasite and leukocyte counts per HPF, emphasis is put on predicting the sequence of regions on the TBS (i.e. the states) that gave rise to the actual parasite and leukocyte counts (i.e. the observations). Since a variation in the distribution of parasites and leukocytes in the TBS is suspected, these regions cannot be directly observed, and need to be predicted. Inference in HMMs is often carried out using the expectation-maximization (EM) algorithm [62-64], but examples of Bayesian estimation implemented through Markov chain Monte Carlo (MCMC) sampling are also frequent in the literature [65,66]. In most practical cases, the number of hidden states is unknown and has to be estimated. The authors shall return to the latter point later in the discussion.

### Methodology

Firstly, the problem of testing whether the data come from a single Poisson distribution is considered. The basic null hypothesis of interest is that “variance = mean” (equidispersion). In a context such as this, the focus is put on alternatives that are overdispersed, in the sense that “variance > mean”. The hypothesis being tested is commonly referred to as the homogeneity hypothesis. A commonly used statistic for testing the Poisson assumption is Pearson’s test, which in spatial statistics is known as the index of dispersion test [67,68]. The statistic is the ratio of the sample variance to the sample mean, multiplied by (***n*-1), where** ***n*** is the sample size.

In the case of the Poisson distribution, the variance is equal to the mean, i.e. the index of dispersion is equal to one. In the case of the binomial distribution, the index of dispersion is less than 1; this situation is called ***underdispersion***. For all mixed Poisson distributions, that show overdispersion in data, the index of dispersion is greater than 1. Fisher [67] showed that under the assumption that data are generated by a Poisson distribution with some parameter ***λ*, then the test statistic approximately has a Chi-squared distribution (*χ***_**2**_**) with (*n***-1) degrees of freedom.

If the Poisson assumption is violated, the goodness of fit of alternative simple parametric models should be assessed. In order to estimate model parameters, a direct optimization of the log-likelihood is performed using optim[69]. The Kolmogorov-Smirnov (k.s) goodness-of-fit test is used [70] to test the validity of the assumed distribution for the data. The test evaluates the null hypotheses (that the data are governed by the assumed distribution) against the alternative (that the data are not drawn from the assumed distribution). Model selection criteria are used to determine which of the simple parametric models best fits the data. The selection criteria used in this paper are presented in the next section.

Secondly, the first source of overdispersion in count data is investigated, which is unobserved heterogeneity. The unobserved heterogeneity among parasite and leukocyte data is explored using mixture models. The motivation behind the use of mixture models is that they can handle situations where a single parametric family is unable to provide a satisfactory model for local variations in data. The objective here is to describe the data as a finite collection of homogeneous populations on TBSs. The form of these sub-populations is modelled using Poisson and NB.

Thirdly, the second source of overdispersion is explored, which is positive contagion [54]. When contagion is present, the value of ***X***_***t***_** positively influences the value of**$X_{t^{\prime }}(t\neq t^{{}^{\prime }})$. For example, a high number of parasites in one HPF leads to correspondingly high numbers of parasites in neighbouring HPFs; likewise, a low number of parasites in one HPF drive down counts for other neighbouring HPFs. Since this data-generating process directly influences the occurrence of parasites in HPFs, it has important implications for the observed level of dispersion in data.

The autocorrelation plots [71] are a commonly-used tool for checking randomness and spatial dependence in data. The autocorrelation function (ACF) will first test whether adjacent observations are autocorrelated; that is, whether there is correlation between observations ***x***_**1**_** and** ***x***_**2**_**,** ***x***_**2**_** and** ***x***_**3**_**,** ***x***_**3**_** and** ***x***_**4**_**, etc. This is known as lag one autocorrelation, since one of the pair of tested observations lags the other by one period (ie. one HPF). Similarly, it will test at other lags. For instance, the autocorrelation at lag five tests whether observations** ***x***_**1**_** and** ***x***_**6**_**,** ***x***_**2**_** and** ***x***_**7**_**,…,** ***x***_**27**_** and** ***x***_**32**_, etc, are correlated. If random, such autocorrelations should be “near zero” for any and all time-lag separations. If non-random, then one or more of the autocorrelations will be significantly non-zero. HMMs are used to account for autocorrelations in data. The state-dependent distribution is modelled using Poisson and NB. Note that HMMs are an extension of mixture models with spatial dependence taken into consideration, and the two types of models are nested.

The proposed mixture models and HMMs are fitted by maximum likelihood using the EM algorithm, and validated by direct numerical maximization using nlm in R[72,73]. Initialization of the EM algorithm is based on incremental k-means [74]. Details on the maximization of the complete-data log-likelihood with regard to parameters of the unobserved state distribution (Poisson, NB) for mixture models and HMMs are given in Additional file 2.

### Model selection and checking

Models comparison was based on three measures. One is the deviance statistic, also called the likelihood-ratio test statistic or likelihood-ratio chi-squared test statistic, which is a measure of the difference in log-likelihood between two models. If data have been generated by Model A (a simpler model) and are analysed with Model B (a more complex model within which model A is nested), the expected distribution of the test statistic, which is twice the difference in log-likelihoods$2({ℒ}_{B}-{ℒ}_{A})$** computed using the data, follows a** ***χ***_**2**_**-distribution with degrees of freedom equal to the difference in the number of parameters. Hence, LRT permits a probabilistic decision as to whether one model is adequate or whether an alternative model is superior. This statistic is appropriate when one model is nested within another model. Negative binomial and Poisson models are nested because as** ***ϕ* converges to 0, the negative binomial distribution converges to Poisson. But the situation is non-standard, because under the null hypothesis the extra parameter** ***ϕ* lies on the boundary of its parameter space. The standard asymptotic result of a** ***χ***_**2**_-distribution is not applicable. For this purpose, Akaike’s Information Criterion (AIC) [75] and the Bayesian Information Criterion (BIC) [76] are used. These two measures penalize for model complexity and permit comparison of nonnested models. Models are nonnested if there is no parametric restriction on one model that produces the second model specification. The AIC (resp. BIC) can be thought of as the amount of information lost when a specific model to approximate the real distribution of data is being used. Thus, the model with the smallest AIC (resp. BIC) is favored.

In the area of statistical modelling (e.g: regression, generalised linear models), residuals are broadly used to check the validity of the fitted model. In this context, residuals are calculated from the model predictions and the observed data. In the context of HMMs, no strict analog to a residual exists since the value of a residual depends on the unobservable state. Pseudo-residuals offer a convenient way for model checking in HMMs [77,78]. The HMM version of residuals is used to check the validity of the model as well as to identify outliers, since their absolute value indicate the deviation from the median of the distribution. While information criteria for model selection compare the relative goodness-of-fit, the analysis of pseudo-residuals provides a measure of the absolute goodness-of-fit. Zucchini and MacDonald [77] provide details for calculating and assessing two types of pseudo-residuals (ordinary and forecast), for both continuous and discrete state distributions. Model pseudo-residuals can also be extracted using the function “Residuals” in the R package HiddenMarkov. Here, the ordinary pseudo-residuals are used to evaluate the suitability of selected HMMs. The ordinary pseudo-residual for the observation ***x***_***t***_** is based on its conditional distribution given all other data. In the case of discrete observations, pseudo-residuals are defined as intervals**$\left[r_{t}^{-},r_{t}^{+}\right]$ as

$$\begin{array}{lll} r_{t}^{-} & =\Phi^{-1}\left(P(X_{t}\;<\;x_{t}|x_{t-1},x_{t-2},\ldots ,x_{1})\right)\;\;\forall t\in ⟦1;T⟧\; & \;\\ r_{t}^{+} & =\Phi^{-1}\left(P(X_{t}\;\leq \;x_{t}|x_{t-1},x_{t-2},\ldots ,x_{1})\right)\;\;\forall t\in ⟦1;T⟧\; & \; \end{array}$$

where ***Φ*** is the c.d.f. of a standard normal-distributed random variable. If the fitted model is correct, the pseudo-residuals are standard normal-distributed. Graphically, QQ-plots and pseudo-residual ACFs were used to assess the goodness-of-fit of selected HMMs.

## Results

### Overdispersion in parasite and leukocyte distributions

Histograms in Figure 1 show that parasite and leukocyte counts are clearly skewed to the right. The fitted “candidate” distributions, Poisson and NB, are displayed on the top of each histogram and compared to the empirical density function in order to visualize how well they match the data. The Poisson distribution clearly does not fit the data. On the other hand, the NB distribution fits the data much more closely than the Poisson distribution. This result was expected because of the implicit restriction of the Poisson model on the distribution of the observed counts. It is true that the negative binomial distribution converges to the Poisson distribution, but the former will be always more skewed to the right than the latter with similar parameters.

The initial visualization of the histograms motivates the use of Pearson’s test to check for overdispersion. In all TBSs, the Poisson model was highly significantly rejected in favor of a model with heterogeneity (***p*** **≪** ***.*** **0001 using Pearson’s test). The authors considered fitting data to alternative models allowing for overdispersion: NB, geometric, logistic, Gaussian, exponential. The k.s test was significant (*p*** **≪** ***.*** 0001), then it indicated that the distribution of the parasite and leukocyte data was significantly different from the distribution against which it was being compared. However, this test is frequently found to be too sensitive. Given a large enough sample size, it can detect differences that are meaningless to the present purpose, in the sense that even very small divergences of the model from the data would be flagged up and cause significance of the test. It is certainly worth judging the results of the test in light of other statistical measures. The AIC is used to assess the goodness-of-fit of alternative models to data. The difference in fit between the Poisson model (resp. NB model) and its corresponding ZIP and HP models (resp. ZINB and HNB models) is trivial. This result might be expected due to the non-excess of zeros in data (see Table 1). The AIC selects the NB model, which is estimated to be “closest” to the unknown distribution that generated the data (△AIC≫10) (see Table 2).

**Table 2:** Comparison of simple parametric models fitted to parasite and leukocyte counts per field

	**Poisson**			**Negative Binomial**		
	-ℒ	**AIC**	**BIC**	-ℒ	**AIC**	**BIC**
***p***_***a***_	6801.59	13605.17	13609.80	3200.63	**6405.25**	**6414.50**
***p***_***b***_	10838.95	21679.91	21684.75	4344.27	**8692.54**	**8702.23**
***p***_***c***_	2472.18	4946.36	4951.08	2302.96	**4609.92**	**4619.38**
***ℓ***_***a***_	3108.25	6218.51	6223.13	2532.77	**5069.53**	**5078.79**
***ℓ***_***b***_	3547.53	7097.06	7101.90	2965.34	**5934.69**	**5944.38**
***ℓ***_***c***_	3051.08	6104.15	6108.88	2728.46	**5460.91**	**5470.37**
	**Geometric**			**Logistic**		
	-ℒ	**AIC**	**BIC**	-ℒ	**AIC**	**BIC**
***p***_***a***_	3249.22	6500.44	6505.06	3287.80	6579.60	6588.86
***p***_***b***_	4413.13	8828.26	8833.10	4407.19	8818.38	8828.06
***p***_***c***_	2488.96	4979.93	4984.65	2344.83	4693.66	4703.12
***ℓ***_***a***_	2719.04	5440.09	5444.72	2560.46	5124.92	5134.17
***ℓ***_***b***_	3122.84	6247.69	6252.53	2998.50	6001.01	6010.69
***ℓ***_***c***_	3122.55	6247.11	6251.84	2762.37	5528.74	5538.20
	**Gaussian**			**Exponential**		
	-ℒ	**AIC**	**BIC**	-ℒ	**AIC**	**BIC**
***p***_***a***_	3279.99	6563.99	6573.24	3248.74	6499.48	6504.10
***p***_***b***_	4384.43	8772.85	8782.54	4412.85	8827.71	8832.55
***p***_***c***_	2327.71	4659.41	4668.87	2482.13	4966.25	4970.98
***ℓ***_***a***_	2560.19	5124.39	5133.64	2717.17	5436.34	5440.96
***ℓ***_***b***_	2995.11	5994.21	6003.90	3118.89	6239.77	6244.62
***ℓ***_***c***_	2765.26	5534.51	5543.97	3120.93	6243.86	6248.59

Parasite (***p***_***a***_**,** ***p***_***b***_**,** ***p***_***c***_**) and leukocyte (*ℓ***_***a***_**,** ***ℓ***_***b***_**,** ***ℓ***_***c***_**) counts are fitted to Poisson, Negative Binomial, Geometric, Logistic, Gaussian and Exponential models. Minus log-likelihood (**-ℒ) and information measures (AIC and BIC) are given. Direct optimization of the log-likelihood was performed using optim in R. The best AIC and BIC values are highlighted in bold.

The maximum likelihood estimators (MLE) for the dispersion parameter of the negative binomial models (***ϕ*) are:**${\hat{\phi }}_{\text{MLE}}(p_{a})=0.53,{\hat{\phi }}_{\text{MLE}}(p_{b})=0.53,{\hat{\phi }}_{\text{MLE}}(p_{c})=0.18,{\hat{\phi }}_{\text{MLE}}(\ell_{a})=0.23,{\hat{\phi }}_{\text{MLE}}(\ell_{b})=0.28,{\hat{\phi }}_{\text{MLE}}(\ell_{c})=0.12$(the maximum likelihood equations are solved iteratively). The positivity of the dispersion parameter of the negative binomial models indicates that parasites (resp. leukocytes) tend to be aggregated together, leaving some areas with high parasite (resp. leukocyte) densities, and other areas with very few parasites (resp. leukocytes) [79]. These findings indicate that there is significant overdispersion in the distribution of parasites and leukocytes across all TBSs used in the analysis.

### Modelling heterogeneity in parasite and leukocyte data

Mixture models fitted to parasite and leukocyte counts are presented in Table 3. Using a two-state Poisson mixture instead of a one-state Poisson model dramatically improved the fit to data as judged by the AIC and BIC contrary to NB case. The simple parametric NB model was preferred to NB mixtures. The goodness-of-fit of Poisson mixtures increased with ***m*** values. Poisson mixtures (slightly) outperformed the one-state NB model according to AIC for TBSs “a” and “b”. However, the one-state NB model was preferred to the Poisson mixtures according to BIC for all TBSs.

**Table 3:** Comparison of independent mixture models fitted to parasite and leukocyte counts by AIC and BIC

	**Poisson mixture**			**Negative binomial mixture**		
***m*** **=1**	-ℒ	**AIC**	**BIC**	-ℒ	**AIC**	**BIC**
***p***_***a***_	6801.59	13605.17	13609.80	3200.63	6405.25	6414.50
***p***_***b***_	10838.95	21679.91	21684.75	4344.27	8692.54	8702.23
***p***_***c***_	2472.18	4946.36	4951.08	2302.96	4609.92	4619.38
***ℓ***_***a***_	3108.25	6218.51	6223.13	2532.77	5069.53	5078.79
***ℓ***_***b***_	3547.53	7097.06	7101.90	2965.34	5934.69	5944.38
***ℓ***_***c***_	3051.08	6104.15	6108.88	2728.46	5460.91	5470.37
***m*** **=2**	-ℒ	**AIC**	**BIC**	-ℒ	**AIC**	**BIC**
***p***_***a***_	3962.18	7930.35	7944.23	3200.63	6409.25	6430.53
***p***_***b***_	5882.41	11770.81	11785.34	4344.27	8696.54	8718.69
***p***_***c***_	2289.73	4585.47	4599.65	2302.96	4613.93	4635.61
***ℓ***_***a***_	2633.87	5273.75	5287.62	2532.77	5073.54	5094.81
***ℓ***_***b***_	3029.67	6065.33	6079.86	2965.35	5938.69	5960.84
***ℓ***_***c***_	2756.98	5519.97	5534.15	2728.45	5464.91	5486.59
***m*** **=3**	-ℒ	**AIC**	**BIC**	-ℒ	**AIC**	**BIC**
***p***_***a***_	3397.75	6805.50	6828.63	3200.63	6413.25	6447.60
***p***_***b***_	4761.19	9532.38	9556.60	4344.27	8700.54	8736.20
***p***_***c***_	2288.39	4586.77	4610.41	2302.96	4617.93	4652.89
***ℓ***_***a***_	2527.85	5065.70	5088.83	2532.77	5077.54	5111.88
***ℓ***_***b***_	2945.87	5901.74	5925.95	2965.35	5942.69	5978.35
***ℓ***_***c***_	2729.21	5468.42	5492.06	2728.45	5468.90	5503.87
***m*** **=4**	-ℒ	**AIC**	**BIC**	-ℒ	**AIC**	**BIC**
***p***_***a***_	3267.46	6548.92	6581.29	3189.16	6394.32	6442.42
***p***_***b***_	4470.16	8954.33	8988.24	4344.27	8704.54	8754.38
***p***_***c***_	2288.21	4590.42	4623.52	2302.96	4621.93	4670.85
***ℓ***_***a***_	2519.22	5052.44	5084.81	2532.77	5081.54	5129.63
***ℓ***_***b***_	2938.52	5891.05	5924.95	2965.35	5946.69	5996.53
***ℓ***_***c***_	2721.23	5456.47	5489.57	2728.45	5472.90	5521.82

Parasite (***p***_***a***_**,** ***p***_***b***_**,** ***p***_***c***_**) and leukocyte (*ℓ***_***a***_**,** ***ℓ***_***b***_**,** ***ℓ***_***c***_**) counts are fitted to Poisson mixtures and negative binomial mixtures. The number of components is** ***m*. Minus log-likelihood (**-ℒ) and information measures (AIC and BIC) are given. Models were fitted by maximum likelihood using the expectation-maximization (EM) algorithm, and validated by direct numerical maximization using nlm in R.

**Figure 2:**
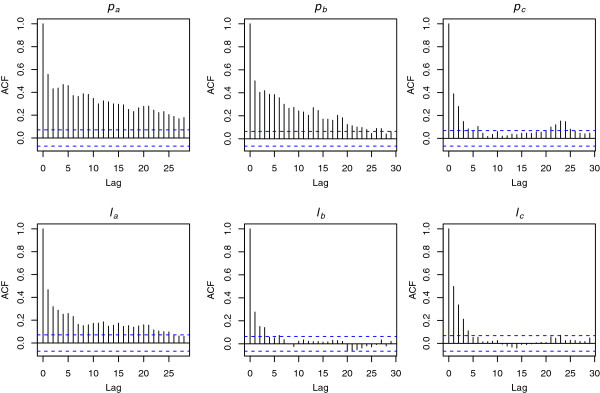
***Sample autocorrelation function (ACF)*.** Autocorrelation plots for parasite (***p***_***a***_**,*p***_***b***_**,*p***_***c***_**) and leukocyte (*ℓ***_***a***_**,*ℓ***_***b***_**,*ℓ***_***c***_**) counts show correlations between values*x***_***i***_** and lagged values of the counts for lags from 0 to 30. The lagged values can be written as*x***_***i*-1**_,***x***_***i*-2**_,***x***_***i*-3**_, and so on. ACF gives correlations between***x***_***i***_** and*x***_***i*-1**_,***x***_***i***_** and*x***_***i***-2_, and so on. The lag is shown along the x-axis, and the autocorrelation is on the y-axis. The blue dotted lines indicate bounds for statistical significance.

Spatial dependence between data is explored through autocorrelation plots (see Figure 2). Autocorrelations should be near-zero for randomness, which was not the case for parasite and leukocyte data. Thus, the randomness assumption failed as expected. The confidence limits are provided to show when ACF appears to be significantly different from zero. Lags having values outside these limits (shown as blue dotted bars) should be considered to have significant correlations. For “***p***_***a***_**”, “*p***_***b***_**” and “*ℓ***_***a***_**”, the autocorrelation plots start with a moderate autocorrelation at lag 1 (between 0.5 and 0.6) that gradually decreases. The decreasing autocorrelation is generally linear, but with significant noise. Such a pattern is the autocorrelation plot signature of a “moderate autocorrelation”, which in turn provides moderate predictability if modelled properly. For parasite data “*p***_***c***_**”, a very few lags >4 slightly lie outside the 95% confidence limits. For leukocyte data “*ℓ***_***b***_**” and “*ℓ***_***c***_”, with the exception of lags <5, almost all of the autocorrelations fall within the 95% confidence limits. For all TBSs, the ACF suggests the existence of a spatial dependence between data. HMMs are therefore used to account for this dependence.

The comparison of independent mixture models in Table 3 and HMMs in Table 4 shows that, on the basis of AIC and BIC, HMMs are superior to mixture models. Although more parameters need to be evaluated for HMMs than for comparable independent mixtures, the corresponding AIC and BIC were lower than those obtained for the independent mixtures. Given the spatial depedence shown in Figure 2, one would expect that independent mixture models will not perform well relative to HMMs.

**Table 4:** Comparison of hidden Markov models fitted to parasite and leukocyte counts by AIC and BIC

	**Poisson HMM**			**Negative binomial HMM**		
***m*** **=1**	-ℒ	**AIC**	**BIC**	-ℒ	**AIC**	**BIC**
***p***_***a***_	6801.59	13605.17	13609.80	3200.63	6405.25	6414.50
***p***_***b***_	10838.95	21679.91	21684.75	4344.27	8692.54	8702.23
***p***_***c***_	2472.18	4946.36	4951.08	2302.96	4609.92	4619.38
***ℓ***_***a***_	3108.25	6218.51	6223.13	2532.77	5069.53	5078.79
***ℓ***_***b***_	3547.53	7097.06	7101.90	2965.34	5934.69	5944.38
***ℓ***_***c***_	3051.08	6104.15	6108.88	2728.46	5460.91	5470.37
***m*** **=2**	-ℒ	**AIC**	**BIC**	-ℒ	**AIC**	**BIC**
***p***_***a***_	3877.14	7764.27	7787.40	3043.31	6098.62	6126.37
***p***_***b***_	5794.89	11599.77	11623.99	4166.23	8344.45	8373.51
***p***_***c***_	2228.73	4467.47	4491.11	2224.71	4461.42	4489.79
***ℓ***_***a***_	2578.83	5167.66	5190.79	2433.86	4879.72	4907.47
***ℓ***_***b***_	2993.67	5997.35	6021.57	2889.88	5791.76	5820.82
***ℓ***_***c***_	2667.70	5345.41	5369.05	2640.61	5293.22	5321.59
***m*** **=3**	-ℒ	**AIC**	**BIC**	-ℒ	**AIC**	**BIC**
***p***_***a***_	6447.60	3265.54	6553.09	6603.97	3008.87	6035.74
***p***_***b***_	4634.75	9291.50	9344.78	4126.32	8270.64	8314.23
***p***_***c***_	2210.74	4443.48	4495.49	2215.95	4449.90	4492.46
***ℓ***_***a***_	2414.70	4851.41	4902.28	2394.82	4807.64	4849.27
***ℓ***_***b***_	2898.08	5818.17	5871.45	2884.03	5786.06	5829.65
***ℓ***_***c***_	2609.50	5241.00	5293.01	2619.57	5257.14	5299.69
***m*** **=4**	-ℒ	**AIC**	**BIC**	-ℒ	**AIC**	**BIC**
***p***_***a***_	3096.91	6231.82	6319.70	2985.36	5994.73	6050.23
***p***_***b***_	4322.77	8683.53	8775.57	4117.57	8259.14	8317.27
***p***_***c***_	2206.93	4451.87	4541.71	2214.22	4452.45	4509.19
***ℓ***_***a***_	2380.19	4798.38	4886.26	2390.87	4805.74	4861.24
***ℓ***_***b***_	2880.72	5799.44	5891.48	2881.97	5787.95	5846.07
***ℓ***_***c***_	2599.52	5237.05	5326.89	2615.98	5255.96	5312.71

Parasite (***p***_***a***_**,** ***p***_***b***_**,** ***p***_***c***_**) and leukocyte (*ℓ***_***a***_**,** ***ℓ***_***b***_**,** ***ℓ***_***c***_**) counts are fitted to Poisson HMMs and negative binomial HMMs. The number of components is** ***m*. Minus log-likelihood (**-ℒ) and information measures (AIC and BIC) are given. Models were fitted by maximum likelihood using the expectation-maximization (EM) algorithm, and validated by direct numerical maximization using nlm in R.

**Figure 3:**
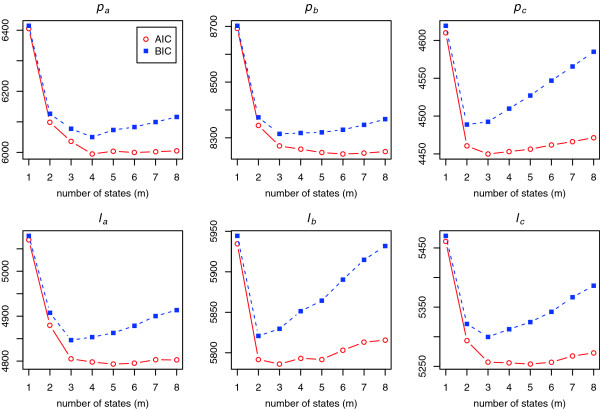
***Model selection criteria of the fitted NB-HMMs*.** AIC and BIC are plotted against the number of states***m* of the negative binomial HMMs fitted to parasite (*p***_***a***_**,*p***_***b***_**,*p***_***c***_**) and leukocyte (*ℓ***_***a***_**,*ℓ***_***b***_**,*ℓ***_***c***_) counts.

Due to its higher complexity, an ***m*-state model will always have a higher likelihood than an (*m*-1)-state model. Model selection criteria are used to see if the improvement in the likelihood was great enough to indicate that the** ***m*-state model captures more heterogeneity in data than the (*m*-1)-state model. Both AIC and BIC, try to identify a model that optimally balances model fit and model complexity. These two criteria are plotted against the number of states** ***m*** of the negative binomial HMM in Figure 3. Several comments arise from Figure 3. Unlike the NB mixtures, using two-state NB-HMM instead of one-state NB-HMM dramatically improves the fit to data. Little to no improvement in AIC is gained for ***m*** **≥3. According to both AIC and BIC, the model with four states is the most appropriate for** ***p***_***a***_**. For the other counts, AIC and BIC selected different models. The Optimal numbers of states selected by LRT (*p*** **≪** ***.*** 0001), AIC and BIC are given in Table 5. AIC and LRT selected the same models. Models selected by AIC and LRT are more complex than those selected by BIC since BIC penalizes larger models more. As it turns out, there is no clear “best” final model. One can narrow down his decision to the two selected NB-HMMs or investigate whether BIC, which selected a smaller “best” model, is more appropriate than AIC in this situation. This would be hard to pin down without extra-statistical information (scientific or practical). It should be noted, however, that the BIC increases consistently after a minimum is attained, while the AIC is more flat around the minimum. This evidence weighs in favour of the BIC.

**Table 5:** Selection of the number of states of the fitted NB-HMMs

	***p***_***a***_	***p***_***b***_	***p***_***c***_	***ℓ***_***a***_	***ℓ***_***b***_	***ℓ***_***c***_
LRT	4	6	3	5	3	5
AIC	4	6	3	5	3	5
BIC	4	3	2	3	2	3

Three selection criteria (LRT, AIC and BIC) were used to select the optimal number of states of the negative binomial HMMs fitted to parasite (***p***_***a***_**,** ***p***_***b***_**,** ***p***_***c***_**) and leukocyte (*ℓ***_***a***_**,** ***ℓ***_***b***_**,** ***ℓ***_***c***_) counts.

**Figure 4:**
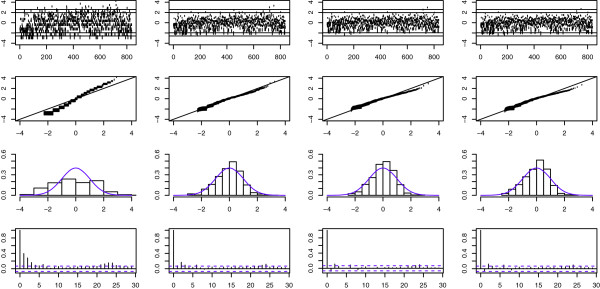
***Diagnostic plots based on normal ordinary pseudo-residuals*.** Rows correspond to (1) index plots of the normal pseudo-residuals with horizontal lines at ±1***.*96 (2.5% and 97.5%) and ±2*.*58 (0.5% and 99.5%), (2) histograms of the normal pseudo-residuals with normal distribution curves in blue, (3) QQ-plots of the normal pseudo-residuals with theoretical quantiles on the horizontal axis, and (4) autocorrelation functions of the normal pseudo-residuals. Columns correspond to the Poisson-HMMs fitted to*p***_***c***_ data with 1, 2, 3 and 4 states respectively.

Even though the AIC and BIC selected two or three-state NB-HMMs for the parasite data ***p***_***c***_, one may consider the Poisson-HMMs as an acceptable alternative, since its AIC and BIC scores were only marginally higher than the competing models (△AIC<10 and △BIC<10). The latter has the advantage of being computationally tractable, while the NB-HMM is more complex as shown in Additional file 2 (higher number of parameters, no analytical solution for the MLE). Hence, one may check whether the Poisson-HMMs provides an adequate fit for the parasite data ***p***_***c***_ using pseudo-residuals. Figure 4 shows that the single Poisson distribution is definitely not appropriate since the pseudo-residuals deviate substantially from the standard normal distribution. In addition, many pseudo-residuals segments lie outside the bands of 0.5% and 99.5%. For the other models, very few observations stand out as extreme, histograms of pseudo-residuals are approximately normal-shaped and autocorrelations are “near zero” indicating low correlation in the residuals. However, the QQ-plots show that the upper quantiles are badly represented for the three and four-state Poisson-HMMs. Considering only the diagnostic plots, and not the model selection criteria, one can accept the two-state Poisson-HMM as the final fitting model for ***p***_***c***_.

## Discussion

The Poisson formulation is seductive in its simplicity. It captures the discrete and nonnegative nature of count data, and naturally accounts for heteroscedastic and skewed distributions through its equidispersion property [80]. However, in most real data situations, equidispersion rarely occurs. The primary objective of the analysis reported in this paper was to test overdispersion in the distribution of parasites and leukocytes per HPF. Pearson’s test was used to test for overdispersion in data. The data are shown to have too much variability to be represented by the Poisson distribution. The primary focus is on fitting the appropriate alternative model to parasite and leukocyte data. The goodness-of-fit of alternative models, designed to address the problem of overdispersion, is illustrated and discussed. The results show that the negative binomial (NB) model is the most appropriate (among simple parametric models), which suggests that parasites and leukocytes tend to aggregate together. The negative binomial has been widely used to inflate the Poisson dispersion as needed [81], and to analyse extra-dispersed count data [82-84]. In addition, typical justifications for using the negative binomial formulation for count data go far beyond the existing critiques of overdispersion. Using the negative binomial distribution instead of the Poisson, allow to fix important errors in model specification [85]. However, both the Poisson and the negative binomial distributions impose some special requirements the credibility of which also needs to be seriously assessed when statistical models for count data are constructed.

To explicitly account for the heterogeneity factor, an alternative model with additional free parameters may provide a better fit. In the case of the parasite and leukocytes counts, the Poisson mixture model and the negative binomial mixture model are proposed. The four-state Poisson model is prefered for two of the three TBSs. In order to further the analysis in the light of the authors’ first intuition (that data tend to aggregate together), autocorrelation plots are examined. ACF suggests the existence of spatial dependence between neighbouring parasite and leukocyte counts. Moreover, investigating sources of overdispersion in data is enhanced by contrasting mixture models to HMMs. On the basis of AIC and BIC, HMMs are prefered. Information from neighbouring regions on TBSs is needed to better estimate this spatial dependence.

In the context of independent mixtures and HMMs, a task of major importance is the choice of the optimal state-dependent distribution and number of states ***m* of the latent process, since the choice of the optimal model leads to the improvement of the goodness-of-fit. The model fit can be increased with increasing** ***m* due to the model likelihood. However, increasing** ***m* increases the number of parameters. Without making assumptions on the transition probability matrix, the problem is quadratic, since the number of parameters is** ***m***^**2**^**+2** ***m*-1 in the case of Poisson-HMMs, and** ***m***^**2**^**+3** ***m***-1 in the case of NB-HMMs.

A compromise has therefore to be found between the model fit and the model complexity. Model selection criteria are used to balance the two situations. They are either based on the full-model log-likelihood (AIC and BIC) [77,86-88], or on reducing the number of parameters by making assumptions on the state-dependent distribution or on the transition probability matrix in the case of HMMs [89,90]. Hypothesis tests, as LRT, can also be used in this context. They have the advantage to allow decisions with a significance level. In this study, LRT and AIC select the same NB-HMMs, which seem to be the best fit for parasite and leukocyte distributions per field on selected TBSs. However, BIC selects less complex NB-HMMs. To the best of the authors’ knowledge, there is no common acceptance of the best criteria for determining the number of states. This issue can best be summarized by a quote from famous Bayesian statistician George Box, who said: ***“All Models are wrong, but some are useful”*** [91].

While it is true that, when fitted to the parasite and leukocyte data, the NB-HMM performed slightly better than the Poisson-HMM on the basis of AIC and BIC, both are reasonable models capable of describing the principal features of the data without using an excessive number of parameters. The NB-HMM perhaps has the advantage to incorporate an extra parameter to allow for overdispersion in parasite and leukocyte counts. However, with small differences in AIC (or BIC) score, i.e: △AIC <10 (or △BIC <10), a statistician may be tempted to choose the Poisson-HMM, which is computationally tractable, rather than its NB counterpart. Either more observations from TBSs or a convincing biological interpretation for one model rather than the other would be needed to take the discussion further. Contrary to the assumptions implicit within widely used simple parametric models, the fit to mixtures and HMMs viewed together are a reflection of the need for an heterogeneous modelling approach that explores the overdispersion in parasite and leukocyte counts.

While at first glance intuitively appealing for a statistician, detecting overdispersion in data is of highly questionable utility for malariologists. From a statistical standpoint, failure to take overdispersion into account leads to serious underestimation of the standard errors, biased parameter estimates and misleading inferences [92]. In addition, changes in deviance (likelihood ratio statistic) will be very large and overly complex models will be selected accordingly. When overdispersion is present and ignored, using the Poisson model may overstate the significance of some covariates [93] or give inconclusive evidence of interactions among them [24]. From an epidemiological point of view, the importance of checking for overdispersion in parasite and leukocyte data stems from the need for epidemiological interpretations to be based on solid evidence. However, most existing PD estimation methods assume homogeneity in the distribution of parasites and leukocytes in TBSs. This assumption clearly does not hold. Likewise, the distribution of blood thickness within the smear will never be completely homogeneous [19], even under optimal conditions. Hence, the validity of the results of many statistical analyses, where PD is related to other explanatory variables, becomes suspect. For example, Enosse ***et al.*** [17] used a Poisson regression to estimate the RTS,S/AS02A malaria vaccine effect, adjusted for parasite density, age, and time to infection. However, the comparison of the analysis outcomes with the primary outcomes of a non-parametric analysis using Mann-Whitney U test appears to show discrepancies. The authors concluded that the Poisson distribution did not adequately describe the data. Another example is the use of logistic regression to model the risk of fever as a continuous function of parasite density in order to estimate the fraction of fever attributable to malaria and to establish a case definition for the diagnosis of clinical malaria [13,15,94]. Case definition for symptomatic malaria is widely used in endemic areas. It requires fever together with a parasite density above a specific threshold. Even under declining levels of malaria endemicity, this method remains the reference method for discriminating malaria from other causes of fever and assessing malaria burden and trends [95]. Such estimates of the attributable fraction may be imprecise if the PD is not being estimated correctly. Furthermore, PD estimation methods potentially induce variability [10]. A proportion of this variability may be explained by the heterogeneity factor. An alternative PD estimation method that accounts for heterogeneity and spatial dependence between parasites and leukocytes in TBSs should be seriously considered in future epidemiological studies with field-collected PD data.

## Authors’ contributions

IH designed and implemented the models, analyzed output data and wrote the manuscript. GN gave technical support and reviewed the manuscript. AG gave conceptual advices and reviewed the manuscript. All authors discussed the results and implications and commented on the manuscript at all stages. All authors read and approved the final manuscript.

## Competing interests

The authors declare that they have no competing interests.

## Supplementary Material

Additional file 1 **Parasite and leukocyte counts per HPF.**The data comprise the records of three TBSs of 12-month-old children from a field study of ***Plasmodium falciparum*** malaria in Tori Bossito, Benin. All HPFs were examined systemically by visually scanning the film horizontally from edge to edge. The numbers of parasites and the number of leukocytes per HPF were recorded. Click here for file

Additional file 2 **EM for mixtures and HMMs.**The statistical tools used to fit the distribution of parasite and leukocyte counts per HPF are presented including the EM algorithm with applications to mixture models and HMMs with Poisson and NB state-dependent distributions. Click here for file
