# Supplementary material for: Evidence for overdispersion in the distribution of malaria parasites and leukocytes in thick blood smears
Source: Malar J. 2013 Nov 6;12:398. doi: 10.1186/1475-2875-12-398 (PMC3831262; doi:10.1186/1475-2875-12-398)
Supplement: Additional file 2 — EM for mixtures and HMMs.The statistical tools used to fit the distribution of parasite and leukocyte counts per HPF are presented including the EM algorithm with applications to mixture models and HMMs with Poisson and NB state-dependent distributions. [file 1475-2875-12-398-S2.PDF]

Evidence for overdispersion in the distribution of malaria parasites and leukocytes in thick blood smears
Additional File 2

Imen Hammami[1], André Garcia[2,3], Grégory Nuel[1]

[1]Laboratoire de Mathématiques Appliquées (MAP5) UMR CNRS 8145,
Université Paris Descartes. Paris, France.
[2]Institut de Recherche pour le Développement,
UMR 216 Mère et enfant face aux infections tropicales.
Université Paris Descartes. Paris, France.
[3]Faculté de Pharmacie, Université Paris Descartes. Paris, France.
E-mail: IH imen.hammami@parisdescartes.fr
AG Andre.Garcia@ird.fr
GN gregory.nuel@parisdescartes.fr

Notation

$X = X_{1:T}$	Observed variables.
X_t	the value of X at time t.
$X_{t:t'}$	Vector of observations $(X_t, ..., X_{t'})$.
$S = S_{1:T}$	Latent (unobserved) variables.
$\Theta^{(k)}$	The estimate of the parameters at iteration k.
$\log P(X \mid \Theta)$	The marginal log-likelihood.
$P(S \mid X, \Theta)$	The posterior distribution.
$\log P(X, S \mid \Theta)$	The complete data log-likelihood (CDLL).
$Q(\Theta \mid \Theta^{(k)})$	The expected CDLL $\sum_S P(S \mid X, \Theta^{(k)}) \log P(X, S \mid \Theta)$.

The EM Algorithm

The expectation-maximization (EM) algorithm is a numerical method for performing maximum likelihood estimation in missing data problems [1].

For a statistical model which is specified through a set of observed data X, a set of unobserved latent data S, and a vector of unknown parameters Θ, along with a likelihood function $L(\Theta \mid X, S) = P(X, S \mid \Theta)$, the maximum likelihood estimate (MLE) of Θ is determined by maximizing the marginal likelihood of the observed data

$$L(\Theta \mid X) = P(X \mid \Theta) = \sum_S P(X, S \mid \Theta) \tag{1}$$

hence

$$\hat{\Theta}_{\text{MLE}} = \arg\max_{\Theta} L(\Theta \mid X)$$

Maximizing $L(\Theta \mid X)$ can be quite tedious because it contains a sum over a large number of S configurations. The EM algorithm allows to circumvent this problem. The algorithm estimates parameters of model Θ that maximize the incomplete data log-likelihood, $\log P(X \mid \Theta)$, by iteratively maximizing the expectation of the complete data log-likelihood, $\log P(X, S \mid \Theta)$. The expected CDLL, with respect to the conditional distribution of S given X, is defined in the EM as an auxiliary function, Q, of current parameter set $\Theta^{(k)}$ and new parameter set Θ given by

$$\begin{aligned} Q(\Theta \mid \Theta^{(k)}) &= \mathbb{E}_{S|X,\Theta^{(k)}}[\log L(\Theta \mid X, S)] \\ &= \sum_S P(S \mid X, \Theta^{(k)}) \log P(X, S \mid \Theta) \end{aligned} \tag{2}$$

If the CDLL is factorizable, optimizing the Q-function could be much easier than optimizing the log-likelihood.

Each iteration consists of an expectation (E) step and a maximization (M) step. After choosing starting values for Θ, the algorithm proceeds as follows :

- **E-step** : Compute $Q(\Theta \mid \Theta^{(k)})$, which gives the conditional expectations of the unobserved data given the observations and given the current estimate of Θ.
- **M-step** : Maximize $Q(\Theta \mid \Theta^{(k)})$, using instead of the unobserved values their conditional expectations from the E-step, that is, solve the optimization problem

$$\Theta^{(k+1)} = \arg\max_{\Theta} Q(\Theta \mid \Theta^{(k)})$$

These two steps are repeated until convergence. A fixed stopping rule determines in advance the desired accuracy of the estimation. For instance, the algorithm may be stopped when $\sum_i \mid \theta_i^{(k)} - \theta_i^{(k-1)} \mid < c$, where c is the convergence criterion. Defining this stopping rule and the starting values for Θ are crucial. The algorithm is conceptually simple and easy to implement. Under mild conditions (e.g. exponential families in [1]), each iteration k of the algorithm is guaranteed to increase the log-likelihood $L(\Theta^{(k)} \mid X)$, and $\Theta^{(k)}$ is guaranteed to converge to a $\hat{\Theta}_{MLE}$.

EM Algorithm for Mixture Models

Mixture models

We assume that X belongs to a heterogeneous population consisting of m homogeneous subpopulations. We assume that, for $t \in [\![1\,;T]\!]$ and $i \in [\![1\,;m]\!]$, X_t is distributed in the ith component with the probability $p_i(X_t \mid \theta_i)$. Let δ_i be the proportion of the ith component, such that $\sum_{i=1}^m \delta_i = 1$. Hence, the marginal probability is

$$P(X_t \mid \Theta) = \sum_{i=1}^{m} \delta_i p_i(X_t \mid \theta_i)$$

The marginal mean of the independent mixture is given by

$$\mathbb{E}(X_t) = \sum_{i=1}^{m} \delta_i \mathbb{E}\left(X_t \mid \theta_i\right)$$

To compute the unconditional variance of the mixture, we use the law of total variance

$$V(X_t) = \mathbb{E}[V(X_t \mid \Theta)] + V[\mathbb{E}(X_t \mid \Theta)]$$

The expected value of conditional variances is given by

$$\mathbb{E}[V(X_t \mid \Theta)] = \sum_{i=1}^{m} \delta_i V\left(X_t \mid \theta_i\right)$$

The variance of the conditional means is given by

$$V[\mathbb{E}(X_t \mid \Theta)] = \sum_{i=1}^{m} \delta_i \mathbb{E}\left(X_t \mid \theta_i\right)^2 - \left(\sum_{i=1}^{m} \delta_i \mathbb{E}(X_t \mid \theta_i)\right)^2$$

In the case of a two-component mixture model with weights δ_i, means μ_i and variances σ_i^2, the total mean and variance will be

$$\mathbb{E}(X_t) = \delta_1\mu_1 + \delta_2\mu_2 \qquad (3)$$

$$V(X_t) = \delta_1\sigma_1^2 + \delta_2\sigma_2^2 + \delta_1\delta_2(\mu_1 - \mu_2)^2 \qquad (4)$$

We will show later that the variance of the mixture model is greater than its expectation, which allows to account for overdispersion in data.

The incomplete-data log-likelihood expression is given by

$$\log L(\Theta \mid X) = \log \prod_{t=1}^{T} P(X_t \mid \Theta) = \sum_{t=1}^{T} \log \left(\sum_{i=1}^{m} \delta_i p_i(X_t \mid \theta_i)\right)$$

The incomplete-data log likelihood may be difficult to maximize. The numerical difficulty is due to the sum inside the log. However, if we assume that observations X are incomplete and that they are generated by an unobserved process S, the likelihood expression can be dramatically simplified, which motivates the use of the EM algorithm.

Before we proceed to the computation of $Q(\Theta \mid \Theta^{(k)})$ in (2), we first need to derive the distribution of the complete data and the distribution of the unobserved data. We have

$$\begin{aligned} \log P(X, S \mid \Theta) &= \sum_{t=1}^{T} \log\left(P(X_t = x_t \mid s_t) P(S_t = s_t \mid \Theta)\right) \\ &= \sum_{t=1}^{T} \sum_{i=1}^{m} \mathbf{1}_{\{s_t = i\}} \log\left(\delta_i p_i(x_t \mid \theta_i)\right) \qquad (5) \end{aligned}$$

and

$$P(S \mid X, \Theta^{(k)}) = \prod_{t=1}^{T} P(s_t \mid x_t, \Theta^{(k)}) \qquad (6)$$

where

$$P(s_t = i \mid x_t, \Theta^{(k)}) = \frac{\delta_i^k p_i(x_t \mid \theta_i^{(k)})}{p(x_t \mid \Theta^{(k)})} = \frac{\delta_i^k p_i(x_t \mid \theta_i^{(k)})}{\sum_{j=1}^{m} \delta_j^k p_j(x_t \mid \theta_j^{(k)})}$$

The E-step

In the context of finite mixtures, the Q-function can be rewritten from (2) using (5) and (6) as

$$\begin{aligned} Q(\Theta \mid \Theta^{(k)}) &= \sum_{S} P(S \mid X, \Theta^{(k)}) \log P(X, S \mid \Theta) \\ &= \sum_{s} \sum_{t=1}^{T} \sum_{i=1}^{m} \mathbf{1}_{\{s_t = i\}} \log\left(\delta_i p_i(x_t \mid \theta_i)\right) P(s_t \mid x_t, \Theta^{(k)}) \\ &= \sum_{i=1}^{m} \sum_{t=1}^{T} \log(\delta_i p_i(x_t \mid \theta_i)) P(s_t = i \mid x_t, \Theta^{(k)}) \\ &= \sum_{i=1}^{m} \sum_{t=1}^{T} \log(\delta_i) P(s_t = i \mid x_t, \Theta^{(k)}) \\ &\quad + \sum_{i=1}^{m} \sum_{t=1}^{T} \log(p_i(x_t \mid \theta_i)) P(s_t = i \mid x_t, \Theta^{(k)}) \qquad (7) \end{aligned}$$

The M-step

Equation (7) can be decomposed in two parts. We maximize the first part with respect to δ_i, the second part with respect to θ_i (λ_i for the Poisson distribution, r_i and π_i for the negative binomial (NB) distribution).

We use a Lagrange multiplier to find the expression of δ_i, since $\sum_{i=1}^{m} \delta_i = 1$. Maximizing the Q-function in (7), subject to the constraint $\sum_{i=1}^{m} \delta_i = 1$, comes down to solving the following equation

$$\frac{\partial}{\partial \delta_i} \left[\sum_{i=1}^{m} \sum_{t=1}^{T} \log(\delta_i) P(s_t = i \mid x_t, \Theta^{(k)}) + \lambda \left(\sum_{i=1}^{m} \delta_i - 1 \right) \right] = 0$$

Then

$$\sum_{t=1}^{T} \frac{1}{\delta_i} P(s_t = i \mid x_t, \Theta^{(k)}) + \lambda = 0 \tag{8}$$

or

$$\sum_{t=1}^{T} P(s_t = i \mid x_t, \Theta^{(k)}) = -\lambda \delta_i$$

Summing over m yields

$$\sum_{i=1}^{m} \sum_{t=1}^{T} P(s_t = i \mid x_t, \Theta^{(k)}) = -\lambda \sum_{i=1}^{m} \delta_i = -\lambda$$

As $\sum_{i=1}^{m} P(s_t = i \mid x_t, \Theta^{(k)}) = 1$, we get $\lambda = -T$.
The maximizing value of δ_i from Equation (8) is

$$\hat{\delta}_i = \frac{1}{T} \sum_{t=1}^{T} P(s_t = i \mid x_t, \Theta^{(k)})$$

M-step for Poisson mixture

Under the Poisson assumption, the maximization is computationally tractable.
Since

$$p_i(x_t \mid \theta^{(k)}) = e^{-\lambda_i} \frac{\lambda_i^{x_t}}{x_t!}$$

differentiating the second term in (7), with respect to λ_i, and equating to zero yields

$$\sum_{t=1}^{T} P(s_t = i \mid x_t, \Theta^{(k)}) \left(-1 + \frac{x_t}{\lambda_i} \right) = 0$$

It follows immediately that

$$\hat{\lambda}_i = \frac{\sum_{t=1}^{T} P(s_t = i \mid x_t, \Theta^{(k)}) x_t}{\sum_{t=1}^{T} P(s_t = i \mid x_t, \Theta^{(k)})}$$

Note that the Poisson mixture model is able to accommodate overdispersion better than the Poisson model with one component. For a two-state Poisson mixture, it follows immediately from (3) and (4) that the variance exceeds the mean by $\delta_1\delta_2(\lambda_1 - \lambda_2)^2$.

M-step for NB mixture

Different parameterizations for the negative binomial distribution exist. We choose the distribution function given by

$$p_i(x_t \mid \Theta^{(k)}) = \frac{\Gamma(x_t + r_i)}{\Gamma(x_t + 1)\Gamma(r_i)} {\pi_i}^{r_i} (1 - \pi_i)^{x_t}$$

where Γ denotes the Gamma-function; $r_i > 0$ and $\pi_i \in [0; 1]$ are the parameters of the NB.

We rewrite the Gamma-functions as $\exp\log(\Gamma)$ in the second part of (7) as follows

$$\begin{aligned} \sum_{i=1}^{m}\sum_{t=1}^{T} P(s_t = i \mid x_t, \Theta^{(k)}) \log(p_i(x_t \mid \theta_i)) &= \sum_{i=1}^{m}\sum_{t=1}^{T} P(s_t = i \mid x_t, \Theta^{(k)}) \\ &\quad (\log\Gamma(x_t + r_i) - \log\Gamma(r_i) - \log\Gamma(x_t) \\ &\quad + r_i \log\pi_i + x_t \log(1 - \pi_i)) \end{aligned} \tag{9}$$

Differentiating (9) with respect to π_i and equating the derivative to zero yields

$$\sum_{t=1}^{T} P(s_t = i \mid x_t, \Theta^{(k)}) \left(\frac{r_i}{\pi_i} - \frac{x_t}{1 - \pi_i} \right) = 0$$

That is

$$\sum_{t=1}^{T} P(s_t = i \mid x_t, \Theta^{(k)})(r_i - \pi_i(r_i + x_t)) = 0$$

The solution is as follows

$$\pi_i = \frac{r_i \sum_{t=1}^{T} P(s_t = i \mid x_t, \Theta^{(k)})}{\sum_{t=1}^{T} P(s_t = i \mid x_t, \Theta^{(k)})(r_i + x_t)} \tag{10}$$

Maximizing (9) with respect to r_i gives

$$\sum_{t=1}^{T} P(s_t = i \mid x_t, \Theta^{(k)}) \left(\psi(r_i + x_t) - \psi(r_i) + \log \pi_i\right) = 0 \tag{11}$$

where

$$\psi(x) = \frac{\partial \log \Gamma(x)}{\partial x} = \frac{\Gamma'(x)}{\Gamma(x)}$$

Substituting π_i from Equation (10) in Equation (11) yields

$$\sum_{t=1}^{T} P(s_t = i \mid x_t, \Theta^{(k)}) \left(\psi(r_i + x_t) - \psi(r_i) + \log \left[\frac{r \sum_{t=1}^{T} P(s_t = i \mid x_t, \Theta^{(k)})}{\sum_{t=1}^{T} P(s_t = i \mid x_t, \Theta^{(k)})(r_i + x_t)}\right]\right) = 0$$

Accurate solution of r_i is obtained with a direct numerical maximization using `optim` in `R` [2]. The maximizing value of r_i is then substituted in (10) to derive π_i.

EM Algorithm for Hidden Markov Models

HMMs

For a sequence data, the assumption of independent samples is too restrictive. The statistical dependence between sets of data may hide critical information. Hidden Markov models (HMMs) are a kind of mixture models where the mixing distribution is a Markov chain, in which, given present, the future is independent of the past. Hidden states are treated as missing data in the estimation of HMM parameters. HMMs are an effective tool for modelling the dependence structure in data.

The model is composed of an observed sequence $\{X_t : t \geq 1\}$ and an unobserved (hidden) sequence $\{S_t : t \geq 1\}$. An observation X_t is generated by a hidden state S_t. Given the state S_t, the observation X_t is independent of other observations and states and only depends on the current state S_t. For a fixed state, the observation X_t is generated according to a fixed probability. If the markov chain $\{S_t : t \geq 1\}$ has m states, $\{X_t : t \geq 1\}$ is called an *m-state* HMM. The process can be drawn as a diagram of states (nodes) and transitions (edges). Figure 1 helps to visualize the architecture of an HMM.

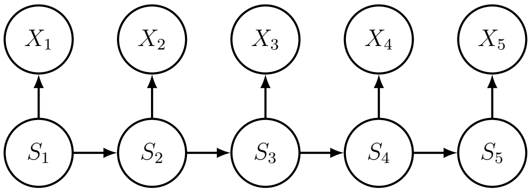

Figure 1: **The basic HMM architecture.** S_1, ..., S_5 are the hidden states and X_1,...,X_5 are the generated observations. Observations are independent of each other and only depend on the current state.

The HMM parameters are transition probabilities and emission probabilities. The transition probabilities $\gamma_{ij} = P(s_t = j \mid s_{t-1} = i)$ control the way the hidden state at time t is chosen given the hidden state at time $t-1$. The process can remain in the state i with probability γ_{ii}. Γ denotes the state transition matrix. The emission probabilities $P(X_t = x_t \mid S_t = i) = p_i(x_t)$ govern the distribution of the observed variable X at time t given the state of the hidden variable at that time.

$\delta_i = P(S_1 = i)$ denotes the initial state probability that specifies the starting state. δ_i may be fixed by specifying a particular state as starting state or under the stationary assumption of HMMs, $\delta = \delta\Gamma$.

We can summarize parameters features by

$$\begin{aligned} P(S_t \mid S_{1:(t-1)}) &= P(S_t \mid S_{t-1}) \quad \forall t \in [\![2\,;T]\!] \\ P(X_t \mid X_{1:(t-1)}, S_{1:t}) &= P(X_t \mid S_t) \quad \forall t \in [\![2\,;T]\!] \end{aligned}$$

The complete-data likelihood is given by

$$P(X, S \mid \Theta) = P(S_1 \mid \Theta) \prod_{t=2}^{T} P(S_{t-1}, S_t \mid \Theta) \prod_{t=1}^{T} P(X_t \mid S_t, \Theta)$$

The CDLL may be straightforward to maximize even if the maximization of the observed data likelihood is tedious. This claim motivates the use of the EM algorithm to fit the HMMs.

Forward and backward probabilities

In order to apply the EM algorithm to HMMs, we need to compute the following probabilities

$$\begin{aligned} \sigma_i(t) &= P(S_t = i \mid X = x) \\ \phi_{ij}(t) &= P(S_{t-1} = i, S_t = j \mid X = x) \end{aligned}$$

To do so, we shall first define the forward probabilities, $\alpha_i(t)$, and the backward probabilities, $\beta_i(t)$.

Definition 1. The forward probability $\alpha_t(i)$ is the probability of the HMM emitting the output symbols $X_{1:t}$, and then ending up in state i at time t.

$$\alpha_t(i) \quad = P(X_{1:t} = x_{1:t}, S_t = i) \quad \forall i \in [\![1\,;m]\!] \quad \forall t \in [\![1\,;T]\!]$$

Definition 2. The backward probability $\beta_t(i)$ is the probability of emitting symbols $X_{(t+1):T}$, then ending up in the final state, given the state at time t is i.

$$\begin{array}{ll} \beta_i(t) & = P(X_{t+1:T} = x_{t+1:T} \mid S_t = i) \quad \forall i \in [\![1\,;m]\!] \quad \forall t \in [\![1\,;T]\!] \\ \beta_i(T) & = 1 \qquad\qquad\qquad\qquad\qquad\quad \forall i \in [\![1\,;m]\!] \end{array}$$

Theorem 1. *Given a state sequence* $\{S_t : t \in [\![1\,;T]\!]\}$ *and an observed sequence* $\{X_t : t \in [\![1\,;T]\!]\}$, *the probability that* X *visits the state* i *at the time* t *is given by*

$$P(X = x, S_t = i) = \alpha_t(i)\beta_t(i) \tag{12}$$

proof 1.

$$\begin{aligned} P(X_{1:T}, S_t = i) &= P(X_{1:t}, X_{(t+1):T}, S_t = i) \\ &= P(X_{1:t}, X_{(t+1):T} \mid S_t = i)P(S_t = i) \\ &= P(X_{1:t} \mid S_t = i)P(X_{(t+1):T} \mid S_t = i)P(S_t = i) \\ &= P(X_{1:t}, S_t = i)P(X_{(t+1):T} \mid S_t = i) \\ &= \alpha_t(i)\beta_t(i) \end{aligned}$$

Proposition 1. *Summing Equation* (12) *over* m *yields*

$$\sum_{i=1}^{m} \alpha_t(i)\beta_t(i) = P(X = x)$$

Theorem 2. *The probability that* X *visited the state* j *at the time* $t-1$ *and enters the state* i *at time* t *is given by*

$$P(X = x, S_{t-1} = i, S_t = j) = \alpha_i(t-1)p_j(x_t)\gamma_{ij}\beta_j(t) \tag{13}$$

proof 2.

$$\begin{aligned}
P(X_{1:T} = x_{1:T}, S_{t-1} = i, S_t = j) &= P(X_{1:t-1}, X_{t:T}, S_{t-1} = i, S_t = j) \\
&= P(X_{1:t-1}, S_{t-1} = i)P(X_{t:T}, S_t = j \mid X_{1:t-1}, S_{t-1} = i) \\
&= P(X_{1:t-1}, S_{t-1} = i)P(X_{t:T}, S_t = j \mid S_{t-1} = i) \\
&= P(X_{1:t-1}, S_{t-1} = i)P(X_t, X_{t+1:T}, S_t = j \mid S_{t-1} = i) \\
&= P(X_{1:t-1}, S_{t-1} = i)P(X_{t+1:T} \mid X_t, S_t = j, S_{t-1} = i) \\
&\quad P(X_t, S_t = j \mid S_{t-1} = i) \\
&= P(X_{1:t-1}, S_{t-1} = i)P(X_{t+1:T} \mid S_t = j) \\
&\quad P(X_t, S_t = j \mid S_{t-1} = i) \\
&= P(X_{1:t-1}, S_{t-1} = i)P(X_{t+1:T} \mid S_t = j) \\
&\quad P(S_t = j \mid S_{t-1} = i)P(X_t = x_t \mid S_t = j) \\
&= \alpha_{t-1}(i)\beta_t(j)\gamma_{ij}p_j(x_t)
\end{aligned}$$

Proposition 2. *$\alpha_t(i)$ is computed by a recursion forward in time.*

$$\alpha_t(i) = \sum_{j=1}^{m} \alpha_{t-1}(j)\gamma_{ji}p_i(x_t)$$

$\beta_t(i)$ is computed by a recursion backward in time.

$$\beta_i(t) = \sum_{j=1}^{m} \beta_i(t+1)\gamma_{ij}p_j(x_{t+1})$$

proof 3. *The proof can be easily deduced from Equation* (12) *and* (13).

$$\begin{aligned}
P(X_{1:T}, S_t = j) &= \sum_{i=1}^{m} P(X_{1:T}, S_{t-1} = i, S_t = j) \\
\alpha_t(j)\beta_t(j) &= \sum_{i=1}^{m} \alpha_{t-1}(i)\beta_t(j)\gamma_{ij}p_j(x_t) \\
\alpha_t(j) &= \sum_{i=1}^{m} \alpha_{t-1}(i)\gamma_{ij}p_j(x_t)
\end{aligned}$$

The same holds for the second recursion.

$$
\begin{aligned}
P(X_{1:T}, S_t = i) &= \sum_{j=1}^{m} P(X_{1:T}, S_t = i, S_{t+1} = j) \\
\alpha_t(i)\beta_t(i) &= \sum_{j=1}^{m} \alpha_t(i)\beta_{t+1}(j)\gamma_{ij}p_j(x_{t+1}) \\
\beta_t(i) &= \sum_{j=1}^{m} \beta_{t+1}(j)\gamma_{ij}p_j(x_{t+1})
\end{aligned}
$$

Proposition 3. *The probability that the process visits the state i at time t given the observed sequence is*

$$
\begin{aligned}
\sigma_i(t) &= P(S_t = i \mid X = x) \\
&= \frac{P(X = x, S_t = i)}{P(X = x)} \\
&= \frac{\alpha_t(i)\beta_t(i)}{P(X = x)} \qquad (14)
\end{aligned}
$$

Proposition 4. *The probability that the process left state i at time $t-1$ and enters state j at t given the observed sequence is*

$$
\begin{aligned}
\phi_{ij}(t) &= P(S_{t-1} = i, S_t = j \mid X = x) \\
&= \frac{P(S_{t-1} = i, S_t = j, X = x)}{P(X = x)} \\
&= \frac{\alpha_{t-1}(i)\gamma_{ij}p_j(x_t)\beta_t(j)}{P(X = x)} \qquad (15)
\end{aligned}
$$

The E-step

The CDLL is given by

$$
\begin{aligned}
\log L(\Theta \mid X, S) &= \log\left(\delta_{s_1} \prod_{t=2}^{T} \gamma_{s_{t-1},s_t} \prod_{t=1}^{T} p_{s_t}(x_t)\right) \\
&= \log(\delta_{s_1}) + \sum_{t=2}^{T} \log \gamma_{s_{t-1},s_t} + \sum_{t=1}^{T} \log p_{s_t}(x_t) \\
&= \sum_{i=1}^{m} \mathbf{1}_{\{s_1=i\}} \log \delta_i + \sum_{i=1}^{m}\sum_{j=1}^{m}\left(\sum_{t=2}^{T} \mathbf{1}_{\{s_t=i,s_{t-1}=j\}}\right) \log \gamma_{ij} \\
&+ \sum_{i=1}^{m}\sum_{t=1}^{T} \mathbf{1}_{\{s_t=i\}} \log p_i(x_t) \qquad (16)
\end{aligned}
$$

Hence

$$\begin{aligned} Q(\Theta \mid \Theta^{(k)}) &= \mathbb{E}_{S|X,\Theta^{(k)}}[\log L(\Theta \mid X, S)] \\ &= \sum_{i=1}^{m} \sigma_i(1) \log \delta_i + \sum_{i=1}^{m} \sum_{j=1}^{m} \left(\sum_{t=2}^{T} \phi_{ij}(t) \right) \log \gamma_{ij} \\ &+ \sum_{i=1}^{m} \sum_{t=1}^{T} \sigma_i(t) \log p_i(x_t) \end{aligned} \tag{17}$$

where $\sigma_i(t)$ and $\phi_{ij}(t)$ are given in (14) and (15).

The M-step

In the M-step, we maximize the CDLL in (16) with respect to the parameter Θ. We maximize the first part with respect to the initial distribution δ_i. We use a Lagrange multiplier to maximize δ_i subject to the constraint $\sum_{i=1}^{m} \delta_i = 1$ as follows

$$\frac{\partial}{\partial \delta_i} \left[\sum_{i=1}^{m} \log(\delta_i)\sigma_i(1) + \lambda \left(\sum_{i=1}^{m} \delta_i - 1 \right) \right] = 0$$

Then

$$\frac{1}{\delta_i}\sigma_i(1) + \lambda = 0 \tag{18}$$

or

$$\sigma_i(1) = -\lambda\delta_i$$

Summing over m yields

$$\sum_{i=1}^{m} \sigma_i(1) = -\lambda \sum_{i=1}^{m} \delta_i = -\lambda$$

As $\sum_{i=1}^{m} \sigma_i(1) = 1$, we get $\lambda = -1$.
The maximizing value of δ_i from Equation (18) is

$$\hat{\delta}_i = \sigma_i(1)$$

We maximize the second part of the CDLL in (16) with regard to γ_{ij} as follows

$$\frac{\partial}{\partial \gamma_{ij}} \left[\sum_{j=1}^{m} \left(\sum_{t=2}^{T} \phi_{ij}(t) \right) \log \gamma_{ij} \right] = 0$$

Equating the derivative to zero yields

$$\frac{\sum_{t=2}^{T} \phi_{ij}(t)}{\gamma_{ij}} - \frac{\sum_{t=2}^{T} \phi_{ii}(t)}{1 - \sum_{k \neq i} \gamma_{ik}} = 0$$

or

$$\frac{\sum_{t=2}^{T} \phi_{ij}(t)}{\gamma_{ij}} - \frac{\sum_{t=2}^{T} \phi_{ii}(t)}{\gamma_{ii}} = 0$$

This implies that

$$\gamma_{ij} \sum_{t=2}^{T} \phi_{ii}(t) = \gamma_{ii} \sum_{t=2}^{T} \phi_{ij}(t)$$

Summing over m gives

$$\gamma_{ii} \sum_{j=1}^{m} \sum_{t=2}^{T} \phi_{ij}(t) = \left(\sum_{j=1}^{m} \gamma_{ij} \right) \sum_{t=2}^{T} \phi_{ii}(t)$$

As $\sum_{j=1}^{m} \gamma_{ij} = 1$, then

$$\hat{\gamma}_{ii} = \frac{\sum_{t=2}^{T} \phi_{ii}(t)}{\sum_{t=2}^{T} \sum_{j=1}^{m} \phi_{ij}(t)}$$

Likewise, the maximizing value of γ_{ij} is then given by

$$\hat{\gamma}_{ij} = \frac{\sum_{t=2}^{T} \phi_{ij}(t)}{\sum_{t=2}^{T} \sum_{k=1}^{m} \phi_{ik}(t)}$$

The maximization of the third part of the CDLL in (16) depends on the nature of the state-dependent distribution (λ_i for the Poisson distribution, r_i and π_i for the negative binomial distribution). For the Poisson distribution, analytic solutions are given. However, the maximization with respect to the parameters is not straightforward under the negative binomial assumption. Hence, numerical maximization is needed. Maximizing values for λ_i and (r_i,π_i) are given in the previous section (EM for mixture models). Note that for HMMs, $P(s_t = i \mid x_t, \Theta^{(k)})$ should be substituted from (14).

References

[1] Dempster, A., Laird, N., Rubin, D.: Maximum likelihood from incomplete data via the EM algorithm. Journal of the Royal Statistical Society **39 (Series B)**, 1–38 (1977)

[2] Nelder, J.A., Mead, R.: A simplex algorithm for function minimization. Computer Journal **7**, 308–313 (1965)
